# Effect of High Fat Dietary Intake during Maternal Gestation on Offspring Ovarian Health in a Pig Model

**DOI:** 10.3390/nu8080498

**Published:** 2016-08-13

**Authors:** Mengmeng Xu, Long Che, Zhenguo Yang, Pan Zhang, Jiankai Shi, Jian Li, Yan Lin, Zhengfeng Fang, Lianqiang Che, Bin Feng, De Wu, Shengyu Xu

**Affiliations:** 1 Key Laboratory of Animal Disease-Resistance Nutrition and Feed Science, Ministry of Agriculture, Sichuan Agricultural University, 211 Huimin Road, Wenjiang District, Chengdu 611130, Sichuan, China; xumengmeng2013@126.com (M.X.); chelong1989@126.com (L.C.); guoguo00002@163.com (Z.Y.); zhangpan5335@sina.cn (P.Z.); shijiankai0227@sina.com (J.S.); lijian522@hotmail.com (J.L.); able588@163.com (Y.L.); fangzhenfeng@hotmail.com (Z.F.); clianqiang@hotmail.com (L.C.); fengb123d@163.com (B.F.); pig2pig@sina.com (D.W.); 2 Key Laboratory of Animal Disease-Resistance Nutrition, Ministry of Education, Sichuan Agricultural University, Wenjiang District, Chengdu 611130, Sichuan, China

**Keywords:** fat level, gestation, offspring, ovary, oxidative stress, apoptosis

## Abstract

Excessive fat intake is a global health concern as women of childbearing age increasingly ingest a high fat diet. We therefore determined the association of a maternal high fat diet in pregnancy with offspring ovarian health during the gestation and postnatal female offspring in pig a model. Thirty-two Yorkshire gilts with similar bodyweights mated at the third estrus were randomly assigned to two nutrition levels of either a control (CON, crude fat: 7.27%) or a high fat diet (HFD, crude fat: 11.78%). Ovary samples were collected during the fetal (Day 55 (g55) and Day 90 of gestation (g90)) and offspring (prepuberty Day 160 (d160) and age at puberty) period to detect ovary development, antioxidant status and apoptosis cells. Maternal HFD did not influence notch signaling gene expression, which regulates primordial follicle formation and transformation, and ovarian histological effect at g55 and g90. However, maternal HFD reduced the numbers of large follicles at d160 and small follicle numbers upon puberty compared to CON in offspring. The results also revealed that the antioxidant index of total antioxidative capability (T-AOC), cytoplasmic copper/zinc superoxide dismutase (CuZn-SOD), glutathione peroxidase (GPx) activities and mRNA expression were higher in the CON than the HFD at g90 and d160, whereas, malondialdehyde (MDA) concentration was decreased in the CON. Maternal HFD increased the inhibitor of the apoptosis-related gene of B-cell lymphoma-2 (*bcl2*) mRNA expression at g90 and d160, whereas, pro-apoptotic-related gene *bcl*-2 assaciated X protein (*bax*) was reduced. These data show that the maternal high fat diet does not delay fetal ovarian development, but it changes ovarian health by the induction of oxidative stress and accelerating cell apoptosis in offspring.

## 1. Introduction

The relationship between nutrition and reproductive health is an active area of research at present [1]. In the Western diet, high fat intake causes metabolic syndromes, including obesity, insulin resistance, type 2 diabetes, hypertension and cardiovascular diseases. Mothers still require a high fat diet in order to ensure a supply of sufficient nutrients to maintain a normal fetal growth trajectory. It has been shown that the offspring of women on a hyperphagia diet that aims to increase fetal birth weight [2] may be at risk of reproductive failure [3]. Meanwhile, evidence in pigs has shown that the reproductive system is tightly regulated by the state of energy reserves [4]. It has been reported that in the female reproductive system, the ovary is a highly sensitive organ [5], and the risk of defective luteal function or reduced follicle numbers is increased by inappropriate nutritional regimens [4]. 

Reproductive health is dependent on the ovarian immune response in humans. The link between oxidative stress and reproductive performance has been well established, with severe oxidative stress rapidly and effectively impairing ovarian development in pigs [6]. Notably, there is an increase in the defective cohort of oocytes in cycling gilts during feeding at high nutritional levels compared to those fed at maintenance levels [7]. Fetal immune function is easily disrupted because of an immature antioxidant defense system. Therefore, it is necessary to investigate the link between high fat nutrition and the fetal immature antioxidant defense system, as well as to study their influence on the reproduction health of offspring.

In porcine ovaries, follicles begin to form at approximately Day 55 of gestation. However, the primary follicle was first detected in pigs at Day 90 of gestation and is rapidly followed by the initiation of follicle growth, such that all stages of pre-antral follicles and antral follicles have been found by age at puberty. All stages of follicles play an important role in ovulation. The antioxidant defense system results in the health progression of all stages of follicle growth and the timing of the onset of ovulation. However, there are few data relating to health changes in the ovary between follicle formation and pubertal ovulation.

The pig is a critical model to investigate the influence of nutrition on ovarian development, because it has biological similarities to human [8]. Therefore, in this study, we examined the effects of nutritionally-induced oxidative stress on follicle numbers, antioxidant-related gene expression, enzymatic activity and cell apoptosis in the ovary. These changes might shed light on the mechanisms mediating the effect of nutrition on reproductive health.

## 2. Materials and Methods

### 2.1. Animals and Diet

All experimental procedures followed the current law regarding animal protection (Ethic Approval Code: SCAUAC201308-2) and were approved by the Guide for the Care and Use of Laboratory Animals prepared by the Animal Care and Use Committee of Sichuan Agricultural University. Thirty-two healthy Yorkshire gilts of similar genetic background and parity were used in this study. Gilts with similar bodyweights (135.54 ± 0.66 kg) were artificially inseminated at the third estrus and were allocated to two nutrition allowance levels, either control (CON, 7.27%) or high fat diet (HFD, 11.78%) levels (*n* = 16/group) (Table 1). The CON diets were formulated to meet nutrient requirements as recommended by the National Research Council 2012 (NRC 2012), and the HFD exchanged fiber that comprised 4.6% of the total diet weight replaced with the same percentage of soy bean oil to increase fat intake by 62%. 

After artificial insemination, the pregnant gilts were housed individually and fed 2 kg/day (0–30 days), 2.4 kg/day (31–90 days) or 3.0 kg/day (91–114 days). Four pregnant gilts from each group were randomly selected at 55 days of gestation (g55) to be anaesthetized with an intravenous injection of Zoletil 50 (0.1 mg/kg body weight; Zoletil 50 Vet, Virbac, Carroscedex, France) and were slaughtered. Four gilts per group were similarly slaughtered at 90 days of gestation (g90). The remaining pregnant gilts were moved to a farrowing room for delivery. After the farrowing of the gilts, the female neonate was defined as having been born (day 1). All female offspring were given ad libitum access to the same diet according to the NRC 2012 (Table 2), and one offspring gilt of close to average weight was randomly selected from each litter and slaughtered at d160 (*n* = 4/group). Upon reaching age at puberty, one gilt of close to average weight was randomly selected from each litter and slaughtered (*n* = 4/group). 

All gilts of the offspring were exposed (with a fence) to mature boars to encourage pubertal estrus. The onset of the estrus and the time of standing heat was determined by an experienced stockperson based on behavioral and vulval characteristics. Gilts standing still under applied back pressure were classified as having experienced puberty onset and defined as age at puberty.

### 2.2. Measurement of Body Weight and Back Fat Thickness

All female fetuses were weighted at g55, g90 and were weighted at birth (1 day). The bodyweights of the gilts of the offspring were determined in the morning following overnight fasting at 160 days and upon puberty. Back fat thickness (BF) of offspring was determined at 6.5 cm from the middle line at the lowest ribs using the LEAN MEATER (RENCO-LEAN MEATER 23249, Renco, Minneapolis, MI, USA). 

### 2.3. Sample Collection

While under anesthesia, both ovaries were collected from all female fetuses of the pregnant pig. One was fixed in 4% paraformaldehyde solution, and the other was rapidly placed in liquid nitrogen and stored at −80 °C until analysis. In detail, in the present study, we selected four gilts from the CON and HFD group at g55 and g90, respectively, and collected all female fetal ovaries; then, we randomly selected two fetal ovary tissues (from two fetuses) from each pregnant gilt used for subsequent mRNA and enzyme activity analysis. At the slaughter of the offspring gilts, both ovaries were removed from each gilt immediately and were washed by PBS three times followed by drying with paper towels. The ovaries were collected for all gilts; one was weighed; the follicle numbers were counted; then, the ovary was fixed in 4% paraformaldehyde solution. The other ovary was rapidly placed in liquid nitrogen and stored at −80 °C until for subsequent RNA extraction and molecular analyses. Follicles were classified as small if they had a diameter of 1–3 mm and were classified as large if they had a diameter of ≥4 mm. The weight of liver and spleen of the offspring gilts was recorded.

Four percent paraformaldehyde fixed ovaries of fetuses and offspring were routinely stained with hematoxylin and eosin. Briefly, samples were dehydrated and embedded in paraffin. Then, samples were cut into 4-μm slices on microtome. Slices were decolorated by dimethylbenzene and dewaxing by ethanol of different concentrations. Then, slices were stained with hematoxylin, hydrochloric acid and eosin. Finally, slices were imaged with a bright field microscope. An individual oocyte surrounded by a single layer of flattened follicular cells was classified as a primordial follicle; primary follicle classification was based on the presence of a single layer of cuboidal follicular cells surrounding an oocyte. Secondary follicles classification was based on the presence of layers of granulosa cells surrounding an oocyte. A follicular antrum surrounded by follicular fluid and cumulus oophorus was classified as an antral follicle.

### 2.4. Gene Expression

To analyze the expression of glutathione reductase (*gr*), glutathione peroxidase (*gpx*), cytoplasmic copper/zinc superoxide dismutase (*CuZn-sod*), manganese superoxide dismutase (*Mn-sod*), extracellular superoxide dismutase (*e-sod*), caspase-3 (*cas3*), bcl-2 assaciated x protein (*bax*) and b-cell lymphoma-2 (*bcl2*), two fetal ovary tissues were randomly selected from each pregnant gilt (*n* = 4), and offspring ovary tissues from each gilt at the prepuberty (160 days) (*n* = 4) and age at puberty stages (*n* = 4) were used to obtain mRNA. Meanwhile, fetal ovary tissues were also used to analyze alpha-1-antitrypsin (*aat*), alpha-fetoprotein (*afp*), 78-kDa glucose-regulated protein (*grp78*), gelsolin (*gen*), LIM homeobox protein 8 (*lhx8*), newborn ovary homeobox gene (*nobox*) and spermatogenesis and oogenesis-specific basic helix-loop-helix 2 (*sohlh2*). Total RNA was extracted from ovary samples using TRIzol reagent (Invitrogen, Carlsbad, CA, USA). Then, reverse transcription was performed using a high-capacity cDNA reverse transcription kit (TaKaRa Biotechnology, Dalian, China) according to the manufacturer’s instructions. The final amplified reaction was performed in a mixture that contained 3 μL of SYBR^®^ Premix Ex Taq™ II, 0.48 μL of the primer pair, 0.12 μL of ROX Reference Dye, 0.6 μL of cDNA template and 1.8 μL of dH_2_O, using a RT-PCR system (ABI 7900HT, Applied Biosystems, Foster City, CA, USA). PCR primers are shown in Table 3. Real-time PCR amplification was conducted with a DNA Engine thermal cycler (PTC-0200, Chromo4 Real-Time Detector; Bio-Rad, Hercules, CA, USA) using thermal cycling conditions: denaturation for 1 min at 95 °C, amplification for 40 cycles with denaturation at 95 °C for 5 s, annealing at 60 °C for 30 s and extension at 72 °C for 30 s and melt curve conditions at 95 °C for 0 s, 50 °C for 30 s and 95 °C for 0 s (temperature change velocity: 0.5 °C/s). Three replicate reactions were performed for each sample, and the 2^−ΔΔCT^ method was used for quantification. The products were electrophoresed on agarose gel to confirm the product size. The identity of each product was confirmed by DNA sequencing. The expression of β-actin has been shown to be consistent and was used as a housekeeping gene to determine the relative transcriptional levels of the target genes in each sample.

### 2.5. Oxidative Stress Biomarkers

Two randomly-selected fetal ovary tissues from each pregnant gilt (*n* = 4) and one ovary tissue from each gilt of the offspring (*n* = 4) were crushed with a mortar and pestle in liquid nitrogen. Commercial assay kits (Nanjing Jiancheng Institute, Nanjing, Jiangsu, China) were used to determine malondialdehyde (MDA), total antioxidative capability (T-AOC), total superoxide dismutase (T-SOD), CuZn-SOD, GR and GPx. Briefly, MDA content and protein concentration were measured based on the thiobarbituric acid (TBA) method, which measures the relative degree of damage to a cell. The activities of SOD, GR and GPx were assayed as described by Jia et al. [9]. All samples were measured in duplicate.

### 2.6. Statistical Analysis

All data are expressed as the mean values and the standard error. Data were analyzed by an independent-samples *t*-test using SPSS 21.0 (IBM SPSS Company, Chicago, IL, USA). All data are shown as the mean ± SEM. *p* < 0.05 was considered significant when used to compare the differences between the CON and the HFD.

## 3. Results

### 3.1. Histological Characteristics in the Ovary of Offspring 

**Figure 1:**
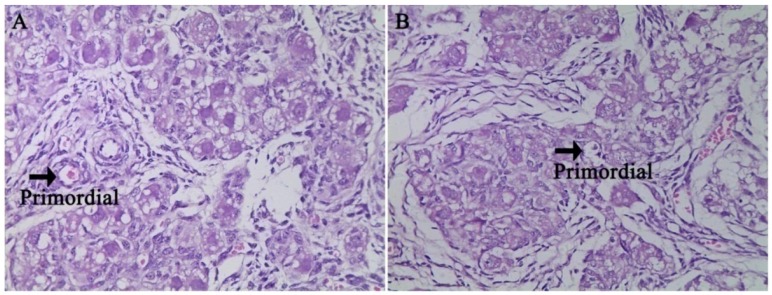
Photomicrograph of ovarian tissue from fetal ovary at Day 55 of gestation with hematoxylin and eosin staining. (**A**) Normal dietary group (CON); (**B**) high fat dietary group (HFD); primordial, primordial follicle. The primordial follicle with both CON (**A**) and HFD (**B**) morphology is identified by black arrows; (**A**) and (**B**) both with original magnification: 400×.

**Figure 2:**
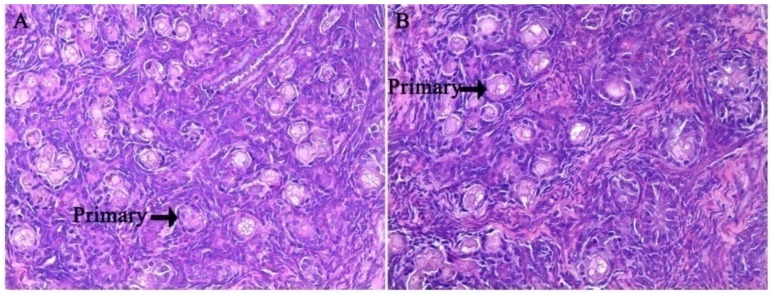
Photomicrograph of ovarian tissue from fetal ovary at Day 90 of gestation with hematoxylin and eosin staining. (**A**) Normal dietary group (CON); (**B**) high fat dietary group; primary, primary follicle. The primary follicle with both CON (**A**) and HFD (**B**) morphology is identified by black arrows; (**A**) and (**B**) both with original magnification: 400×.

**Figure 3:**
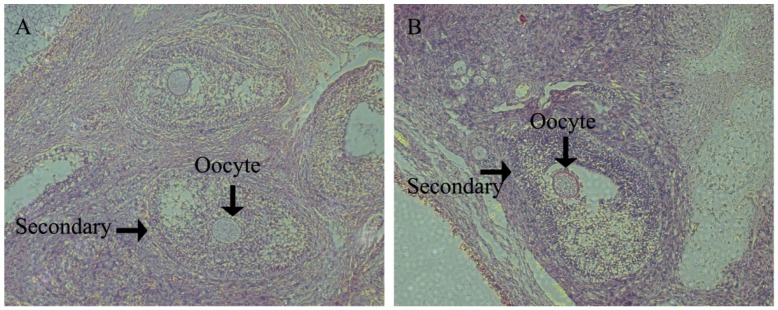
Ovary histological examination of the follicle of the offspring at Day 160 of offspring with hematoxylin and eosin staining. (**A**) Normal dietary group (CON); (**B**) high fat dietary group (HFD); secondary, secondary follicle. The secondary follicle and oocyte with both CON (**A**) and HFD (**B**) morphology are identified by black arrows; (**A**) and (**B**) both with original magnification: 100×.

**Figure 4:**
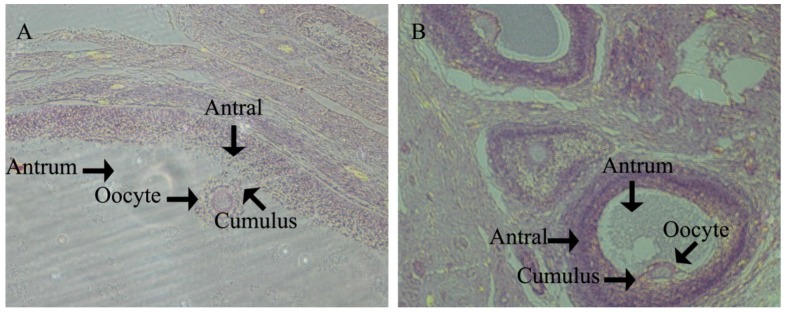
Ovary histological examination in follicle of offspring upon puberty with hematoxylin and eosin staining. (**A**) Normal dietary group (CON); (**B**) high fat dietary group (HFD); antral, antral follicle; cumulus, cumulus oophorus; antrum, follicle antrum. The antral follicle, oocyte, follicle antrum and cumulus oophorus with both CON (**A**) and HFD (**B**) morphology are identified by black arrows; (**A**) and (**B**) both with original magnification: 100×.

There were no significant differences in fetal ovary development at g55 or g90 between the two treatments (Figure 1 and Figure 2). As shown in Figure 1, the primordial follicles are present both in the CON and HFD. The primary follicle of the fetal ovary was detected at g90 in both groups (Figure 2). Those results indicated that maternal high fat diet does not accelerate the process of fetal ovarian development. We also observed that secondary follicles are present both in the CON and HFD (Figure 3) at d160 of offspring, and a large number of antral follicles was detected in both groups for age at puberty (Figure 4).

### 3.2. Reproductive and Growth Performance of Offspring Gilts

As shown in Table 4, pigs’ offspring body weights were not significantly influenced by maternal fat level treatments at all time stages. We also observed that the maternal delivery time was not significantly influenced by maternal fat level treatments (114.75 ± 0.48 vs. 114 ± 0.41, *p* = 0.278). Then, we observed and recorded the numbers of large follicles and small follicles in the ovaries of adult offspring gilts to determine if maternal nutrition could have long-term effects. To evaluate the numbers, we harvested ovaries from the gilts’ offspring at d160 and upon puberty. As shown in Table 5, the offspring of the HFD group had significantly reduced large follicle numbers (*p* < 0.05) compared to the CON group at d160, and the HFD group showed a marked reduction in small follicle numbers for age at puberty (*p* < 0.05).

The liver weight of the offspring was significantly reduced (*p* < 0.05) in the HFD group at d160, but spleen weight was not significantly influenced by maternal fat treatments. Table 5 shows that BF was significantly increased (*p* < 0.05) in the HFD group (*p* < 0.05) compared to the CON group at d160 and upon puberty. However, upon puberty onset, maternal fat level treatments had no significant influence.

### 3.3. Ovarian Oxidative Stress Status

**Figure 5:**
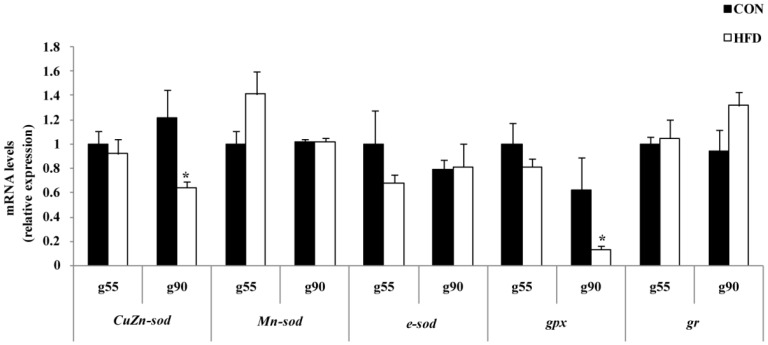
Relative mRNA expressions of antioxidant stress in ovary of fetus. *CuZn-sod*, cytoplasmic copper/zinc superoxide dismutase; *Mn-sod*, manganese superoxide dismutase; *e-sod*, extracellular superoxide dismutase; *gpx*, glutathione peroxidase; *gr*, glutathione reductase. g55, at Day 55 of gestation of the fetus; g90, at Day 90 of gestation of the fetus (*n* = 4 for each group; the samples were from four gilts, and each gilt provided two fetuses; the calculated average values of the two fetal ovaries for each gilt were used in the statistical analysis); * *p* < 0.05. The data were normalized to Gestation Day 55 (g55) of the CON group.

**Figure 6:**
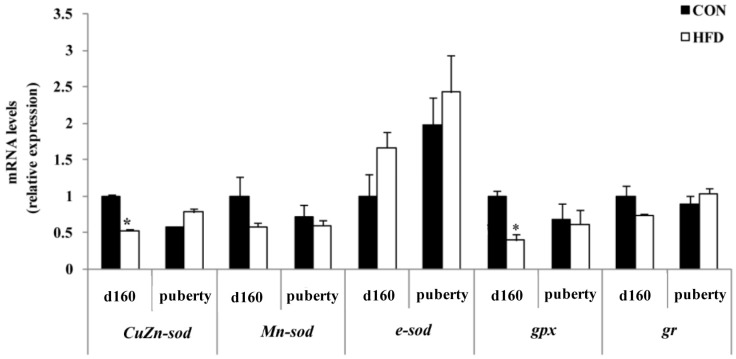
Relative mRNA expressions of antioxidant stress in ovary of offspring. *CuZn-sod*, cytoplasmic copper/zinc superoxide dismutase; *Mn-sod*, manganese superoxide dismutase; *e-sod*, extracellular superoxide dismutase; *gpx*, glutathione peroxidase; *gr*, glutathione reductase, d160 , at Day 160 of offspring; puberty, upon the offspring reaching puberty (*n* = 4 for each group); * *p* < 0.05. The data were normalized to d160 of the CON group.

Based on previous work showing that oxidative stress status was responsible for follicle apoptosis, we investigated the levels of antioxidant enzymes. The oxidative stress status-related genes expressions of the fetus were not altered in either of the treatments at g55 of gestation. As shown in Figure 5, the HFD group had a significant reduction in the expression of *CuZn-sod* and *gpx* at g90. Nevertheless, the mRNA expressions of *Mn-sod*, *e-sod* and *gr* were not significantly affected by maternal fat treatments at g90. To demonstrate that maternal nutrition has important long-term effects on the offspring reproductive health, we tested additional related genes involved in oxidative processes and found that the HFD group had significantly reduced expression of *CuZn-sod* and *gpx* relative to the offspring of the CON group at d160 of offspring (Figure 6). 

Enzyme activity further indicated that the MDA concentration was markedly higher (*p* < 0.05) at g90, but T-SOD, CuZn-SOD, GPx, T-AOC and GR activities were significantly reduced (*p* < 0.05) at g90 in the HFD group compared to the CON group (Table 6). In addition, as shown in Table 6, GPx, T-SOD and T-AOC activity were significantly lower (*p* < 0.05) in the offspring of the HFD group than in the CON group at d160. However, as shown in Table 6, enzymes activities were not significantly influenced by maternal fat treatments at g55 and puberty, except that T-AOC was significantly lower (*p* < 0.05) at g55 in the HFD group compared to in the CON group.

### 3.4. Ovarian Development and Apoptosis 

**Figure 7:**
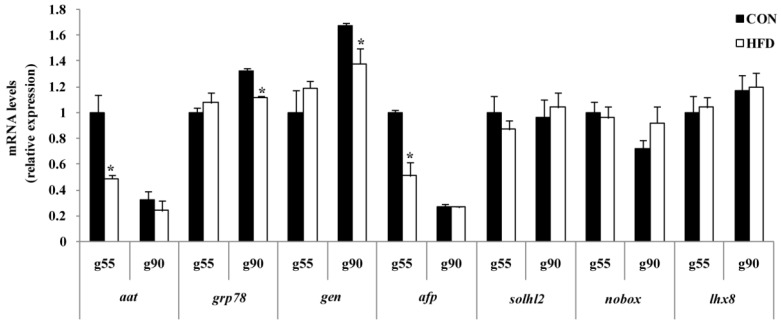
Relative mRNA expressions of follicle formation and development in the ovary of the fetus. g55, at Day 55 of gestation of fetus; g90, at Day 90 of gestation of fetus; *aat*, alpha-1-antitrypsin; *grp78*, 78-kDa glucose-regulated protein; *gen*, gelsolin; *afp*, alpha-fetoprotein; *sohlh2*, spermatogenesis and oogenesis-specific basic helix-loop-helix 2; *nobox*, newborn ovary homeobox gene; *lhx8*, LIM homeobox protein 8 (*n* = 4 for each group; the samples were from four gilts and each gilt provided two fetuses; the calculated average values of the two fetal ovaries for each gilt were used in the statistical analysis); * *p* < 0.05. The data were normalized to g55 of the CON group.

In European porcine ovaries, primordial follicles begin to form at approximately g55, and the primary follicle was first detected at g90. To investigate the pathways involved in primordial follicle formation and transformation to the primary follicle, the mRNA levels of several transcription factors in the notch signaling pathway were examined, including SOHLH2, NOBOX and LHX8, which are critical for the formation and transformation of primordial follicles. The relative mRNA expression levels of *sohlh2*, *nobox* and *lhx8* were not altered in HFD-treated compared to CON in the fetal periods (Figure 7). Then, we tested the levels of gene expression for those that regulate primordial follicle formation and transformation of environmental homeostasis. The results showed that levels of *aat* (*p* < 0.05) and *afp* (*p* < 0.05) mRNA in HFD-treated ovaries were lower compared to the CON group ovaries at g55, and *grp78* (*p* < 0.05) and *gen* (*p* < 0.05) were higher in the CON group relative to the HFD-treated ovaries at g90 (Figure 7). 

**Figure 8:**
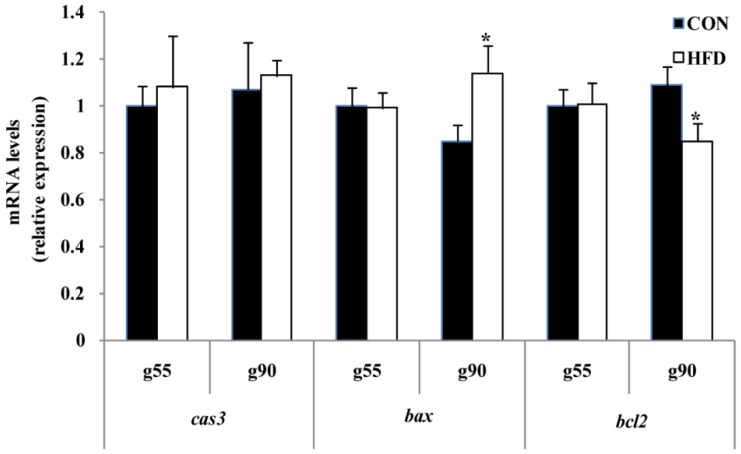
Effect of maternal nutrition on ovary cell apoptosis of fetus. *cas3*, Caspase-3; *bax*, Bcl-2 assaciated X protein; *bcl2*, B-cell lymphoma-2. g55, at Day 55 of gestation of fetus; g90, at Day 90 of gestation of fetus (*n* = 4 for each group; the samples were from four gilts, and each gilt provided two fetuses; the calculated average values of the two fetal ovaries for each gilt were used in the statistical analysis); * *p* < 0.05. The data were normalized to g55 of the CON group.

**Figure 9:**
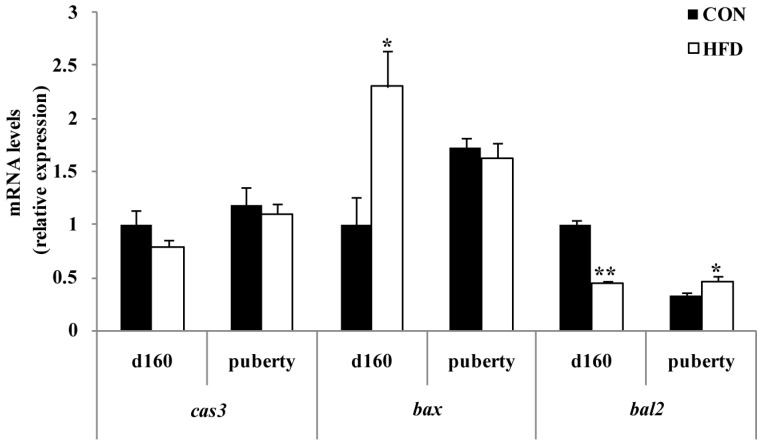
Effect of maternal nutrition on ovary cell apoptosis of offspring gilt. *cas3*, Caspase-3; *bax*, Bcl-2 assaciated X protein; *bcl2*, B-cell lymphoma-2. d160, at Day 160 of offspring; puberty, upon the offspring reaching puberty. (*n* = 4 for each group); * *p* < 0.05; ** *p* < 0.01. The data were normalized to d160 of the CON group.

In order to investigate whether the oxidative stress status was responsible for follicle apoptosis, we tested the mRNA expression of the apoptosis-related gene. Furthermore, the pro-apoptosis-related gene of *bax* was not significantly influenced at g55, but had dramatically higher expression in the HFD-treated group than the CON group at g90. In contrast, the apoptosis suppression factor *bcl2* had dramatically lower expression in ovary cells from HFD-treated animals at g90, as shown by real-time PCR analyses (Figure 8). In addition, *bcl2* was significantly reduced, and *bax* was significantly increased in the HFD group (*p* < 0.05) compared to the CON group at d160 (Figure 9), but *bcl2* was significantly increased in the HFD group (*p* < 0.05) upon puberty of the offspring (Figure 9). The results also revealed that caspase-3 (*cas3*) was not altered in either group at any of the time points (Figure 8 and Figure 9).

## 4. Discussion

Disorders of the reproductive system are a major health issue worldwide in both humans and domestic animals. A few studies have suggested that maternal dietary intake may regulate reproductive outcomes [10,11], at least in part by ovarian oxidative status and cell apoptosis [12]. An appropriate balance of free radicals is critical for the optimal development of the ovary, as free radicals have both beneficial and harmful effects [13]. Meanwhile, fetal ovarian structure, physiology and metabolism may change depending on maternal dietary intake and, therefore, lead to vulnerability to reproductive disorders in adults [14]. In order to explore the molecular mechanisms mediating the long-term effect of maternal dietary intake on the reproductive health in their offspring, the effect of nutrient intake, including oxidative stress and apoptosis, was examined in this study.

Strong evidence from animal studies has shown that dietary manipulations can result in changes in reproductive function [11]. Experimental studies in humans demonstrated that high fat maternal dietary intake during pregnancy may promote a greater neonatal weight [15]. Previous data from our laboratory supports earlier findings that a maternal diet with added soybean oil to increase fat levels achieves a higher neonatal weight [10]. However, in this study, the effect of fat intake does not alter female fetal weight or offspring weight. One reason for this may be that we only selected female fetuses. Fetuses receive constant nutrients from mothers via the placenta; therefore, the mother’s body condition and glucose levels during the gestation period play an important role in the growth of fetuses. Maternal fat intake level not only helps to maintain the pregnancy, but also affects the growth of fetuses. Previous data from our laboratory showed that gilts with increased fat intake of 62% resulted in a significant increase of maternal body weight (201.65 ± 3.60 vs. 188.30 ± 1.82, *p* = 0.035) and BF (18.65 ± 1.20 vs. 16.89 ± 0.96, *p* = 0.005) at g90. Meanwhile, the results showed that a high fat level led to significantly increased maternal glucose level (+49%) and fetuses glucose level (+121%) compared to the CON group at g90. Although maternal glucose levels increase 49%, which would be considered in the pre-diabetic/diabetic range in humans, through self-regulation of glucose levels in gilts, these returned to normal levels after delivery. Furthermore, there is little information in pigs regarding what blood glucose levels might be defined as diabetic. Experimental studies in mice demonstrated upregulation of glucose transport in the placenta, thus potentially linking maternal high fat diet and fetus glucose level [16,17]. Previous unpublished data (2016) from our laboratory showed that gilts with a high fat diet (increased 62% compared to control) increased placental nutrient transport, which led to an increased fetus glucose level [18]. The neonatal (one day) glucose level had increased in the HFD compared to CON (+66%). Therefore, it is possible that maternal fat level may affect the maternal condition and glucose level of fetuses. In this study, the effect of fat intake does not alter female fetal weight and offspring weight. Another reason for this may be that maternal fat intake had a significant influence on reproductive organs instead of body weight. Consistent with this view, it was demonstrated that the effect of maternal fat intake does not alter the glucose level of female offspring at d160 (4.91 ± 0.38 vs. 4.83 ± 0.39) and upon puberty (4.10 ± 0.35 vs. 4.68 ± 0.43) stages.

In swine development, Yorkshire gilts will see the onset of their estrus cycle at Day 180. Therefore, we investigated ovary development at d160 in order to assess the gilts’ reproductive health and to analyze how the follicles within the ovaries of prepubertal gilts appear to form the follicle reserves for future reproductive life. We found that at d160 in the HFD group, gilts had significantly lower large follicle numbers than CON gilts. Meanwhile, HFD group gilts had significantly small follicle numbers upon puberty, which is essential for fertility preservation for adult female animals and beneficial to ovulation. This suggests that ovarian growth potential established prenatally may influence the development of adult reproductive function [19], and maternal fat levels may lead to ovary change. To further confirm that maternal fat level has adverse effects on reproductive function, BF was determined to examine the ponderal index, and we found that it significantly increased in the HFD group compared to the CON group at puberty. This finding suggested that maternal diet affects body index in the offspring. This is consistent with the observation that high BF gilts reach puberty earlier than low BF gilts. Optimum BF was 15–18 mm [20]. If BF exceeds this optimum, it will cause a decline in feed intake during lactation, which will affect the health of the offspring. Moreover, antioxidant defense system damage and an increase in free radicals caused by high BF will lead to feed intake being reduced [21]. Meanwhile, clinical studies have indicated that women with polycystic ovaries tend to have high body weight, and patterns of hormone release in offspring depend on maternal body mass index in pregnancy [14]. In the present study, we found that BF was 19.6 mm in the CON group, which was nearly the optimum BF. In swine development, gilts are usually kept under nutrient restriction to achieve optimum BF of both the gilt/offspring [22]. As the present study was aimed at providing guidance to humans, both the offspring and gilts were fed ad libitum, and this may explain why offspring BF was greater than the optimum BF. This implied that gilts in the CON group will provide a better maternal environment for fetal ovary development and indicated that maternal high nutrient intake would influence the function of ovaries in later adult life. These adverse effects of maternal high nutrient intake are likely to be associated with ovary formation and intrauterine development. 

In the fetal ovaries of pigs, germ cells migrate to the gonad and remain in clusters [23]. The clusters undergo apoptosis and break up, becoming enclosed in primordial follicles consisting of one oocyte and several somatic granulosa cells and transforming into the primary follicle [24]. These are critical processes in ovarian biology [25]. Therefore, in the present study, we observed two critical periods during the formation and transformation of primordial follicles. Early studies found that Notch signaling mediates primordial follicle formation and transformation, particularly involving the SOHLH2, NOBOX and LHX8 transcription factors [26,27]. Early studies found that downregulation of mRNA expression of *sohlh2*, *nobox* and *lhx8* alters primordial follicle numbers and results in a lifelong reduction in the primordial follicle pool [26]. Interestingly, in the present study, we did not observe a difference in the gene expression level of *sohlh2*, *nobox* and *lhx8* in the fetal ovary. Meanwhile, the histological characteristics in the ovary of fetus showed that primordial follicles and primary follicles were not significantly different in the fetus ovary at g55 or g90 between the two groups. Previous studies have shown that *sohlh2*, *nobox* and *lhx8* mRNA expression and proteins are relatively concordant in ovary [26,28]. Thus, the results indicate that maternal nutrition during pregnancy might not delay fetal ovarian development. 

To further confirm whether maternal nutrition affects ovary health and homeostasis, the levels of *aat*, *afp*, *grp78* and *gen* were determined. AAT is a protease inhibitor that is supposed to finely regulate the balance of protease activities and is a biomarker of primordial follicle health [29]. AFP is essential for immune response in ovaries [30]. GRP78 is a molecular chaperone that facilitates protein assembly, folding and regulation of endoplasmic reticulum (ER) stress signaling [31,32,33], and GEN is known to be a cell motility factor [34]. In the present study, mRNA expressions of *aat* and *afp* at g55 and *grp78* and *gen* at g90 were decreased in the HFD group compared to the CON group. Early studies also found that these homeostasis-related genes’ mRNA expression is relatively concordant in ovary with proteins [35,36]. Our previous study has confirmed that *aat* and *afp* might promote primordial follicles formation, and *grp78* and *gen* could protect primordial follicles from transforming to primary follicles [37]. These results demonstrate that the impaired expression of genes regulating homeostasis (*aat*, *afp*, *grp78* and *gen*) in the ovary could be modulated by high fat diets. Although there is no effect of maternal nutrition on ovarian development progress, it may damage its capacity to resist oxidative stress. The findings also indicated that maternal homeostasis reflects the offspring’s health trajectory of growth, and high nutrition levels will result in cell damage and premature follicle pool failure.

To further clarify the effect of maternal fat nutrition on premature follicle pool failure, we examined the expression of factors that regulate cell survival. BCL2 is known to be an anti-apoptotic factor and is important in the survival of ovarian cells [38]. BCL2 can inhibit the formation of lipid peroxides and prevent the production of free radicals [39]. High expression of BCL2 resulted in either higher ovulation rate or litter size [40], and deletion of BCL2 led to complete blockade of follicular oocyte development [41]. BAX opposes BCL2 function and is a pro-apoptotic protein [42]. Previous studies reported that BAX is expressed in both fetal and adult ovaries [43], and BAX expression is increased in atretic follicles compared to healthy ones [44]. In our study, we found that the level of bax was dramatically higher in ovary cells in the HFD group at g90 with BCL2 showing the opposite trend. This further indicated that high fat nutrition accelerates follicle pool exhaustion, which may reduce sow reproduction longevity. To date, many studies have indicated that maternal and placental systems supply sufficient nutrients to ensure fetal growth [16]. Thus, further experiments should include testing of offspring ovary apoptosis-related genes to determine whether maternal nutrition is truly regulating their offspring’s reproductive health throughout the pregnancy period. To investigate this, we examined mRNA expression of *bcl2* and *bax* in the developing ovaries of offspring. We found that the expression of *bcl2* decreased and *Bax* increased significantly with increasing dietary energy levels of the offspring at d160. Interestingly, we also found that *bcl2* significantly increased in the HFD group, but *bax* expression level did not change for age at puberty. This may be due to a temporal effect of maternal fat level on cell survival. Although related protein expression levels were not measured in the present study, previous studies found that *bcl2* and *bax* mRNA expression and protein expression levels are relatively concordant in ovary [45]. The combination of these results may demonstrate a role of maternal fat nutrition in the regulation of apoptotic signaling pathways and mediating the long-term effects on offspring ovary development.

Epidemiologic studies suggested that fetuses that were exposed to an adverse intrauterine environment tended to have relatively high disease rates in adult life [16]. Meanwhile, there was the finding that the long-term effects of adverse free radical imbalance were based on the program of following up with animals in the middle of life whose intrauterine health index measurements had been previously recorded [8]. The imbalance of free radicals could reduce the capability of antioxidant systems, leading to oxidative stress, which can impair organism structure, metabolism and physiology, thereby predisposing individuals to abnormal functionality [16]. In this study, a high fat maternal nutrient supply would satisfy fetal nutrient requirements, but results in a range of fetal maladaptations and may deteriorate the body’s antioxidant defense system. The increased concentration of MDA was supportive of the reduced T-SOD, CuZn-SOD, GPx and GR enzyme activity and gene expression of *CuZn-sod* and *gpx* in the ovary of the HFD group at g90. Our results showed that the mRNA expression and protein expression levels of oxidative stress-related genes are relatively concordant in swine ovary. With plenty of catabolism, the fetus will experience rapid growth in the first Day 90 of pregnancy, leading to an increase in free radicals. Then, maternal fat level affects the related enzyme activity at g90, but no significant influenced is seen at g55. Interestingly, we found that in the HFD group, T-AOC activity was only reduced upon the offspring reaching puberty compared to the CON group of the offspring. It is possible that maternal fat level may lead to a lifelong failure in the offspring’s antioxidant system. However, further ovarian apoptosis of offspring by immunohistochemistry and offspring fertility merit investigation. 

A high fat diet does not alter fetal weight and may induce oxidative stress, as well as accelerate cell apoptosis, which can have adverse effects on the reproductive health of offspring due to their damaged ovaries.

## 5. Conclusions

The findings of the present study show that increasing maternal feed fat levels during gestation results in altered ovarian health of offspring by the induction of oxidative stress and the acceleration of cell apoptosis. Our findings provide new insights into how a high fat diet during pregnancy plays an important role and not only influences the future of ovary apoptosis and anti-oxidative capacity of a child, but also influences the gilts’ follicle health upon the offspring reaching puberty and does not provide an optimal intrauterine environment for the fetus. Future work will be needed to determine an appropriate diet to ensure a reduction in the incidence of disease in the offspring of either humans or gilts. 

## Figures and Tables

**Table 1:** Ingredients and nutrient content of gilt diets.

Ingredient (%)	Pregnancy		Lactation
	HFD	CON	
Corn	45.00	45.00	62.12
Soybean meal	13.60	13.60	23.24
Fish meal	-	-	3.00
Wheat bran	27.80	27.80	5.00
Soy oil	9.10	4.50	2.50
Wheat fiber	-	2.54	-
Soybean fiber	-	1.10	-
Corn fiber	-	0.96	-
Salt	0.40	0.40	0.40
Choline chloride	0.14	0.14	0.15
Calcium carbonate	1.24	1.24	1.10
Dicalcium phosphate	1.99	1.99	1.27
Sodium bicarbonate	-	-	0.40
Lysine	0.10	0.10	0.23
Threonine	0.10	0.10	0.03
Tryptophan	-	-	0.01
Premix	0.53 *	0.53 *	0.55 ^&^
Total	100.00	100.00	100.00
Nutritional compositions, %			
Digestible energy, MJ/kg	3.40	3.00	3.26
Crude fat	11.78	7.27	-
Crude fiber	3.41	4.97	-
Crude protein	13.49	13.92	17.67
Lysine	0.69	0.69	1.07
sulfur-containing amino acid	0.35	0.35	0.55
Threonine	0.46	0.46	0.73
Ca	0.96	0.96	0.90
Total P	0.79	0.79	0.68

* Supplied the following per kilogram of complete diet: 12,000 IU of vitamin A; 3250 IU of vitamin D_3_; 16 IU of vitamin E; 5.2 mg of riboflavin; 20 mg of nicotinic acid; 11 mg of pantothenic acid; 0.12 mg of vitamin B_12_; 0.13 mg of biotin; 170 mg of Fe; 17 mg of Cu; 160 mg of Zn; 35 mg of Mn; 0.3 mg of Se; 0.28 mg of I; ^&^ supplied the following per kilogram of complete diet: 15,100 IU of vitamin A; 3050 IU of vitamin D_3_; 15 IU of vitamin E; 5 mg of riboflavin; 18 mg of nicotinic acid; 12 mg of pantothenic acid; 0.13 mg of vitamin B_12_; 0.14 mg of biotin; 160 mg of Fe; 16 mg of Cu; 80 mg of Zn; 30 mg of Mn; 0.3 mg of Se; 0.20 mg of I. CON, normal dietary group and gilts were fed diets containing 7.27% crude fat during gestation; HFD, high fat dietary group and gilts were fed diets containing 11.78% crude fat during gestation.

**Table 2:** Ingredients and nutrient content of offspring gilt.

Ingredient (%)	5–8 Weeks	9–13 Weeks	14–18 Weeks	19 Weeks–Puberty
Corn	26.70	68.30	70.50	74.80
Extruded corn	27.70	-	-	-
Extruded soybean	13.00	-	-	-
Soybean meal	15.90	24.00	24.74	21.30
Soy oil	0.90	2.00	2.00	1.50
Fish meal	4.50	3.00	-	-
Sucrose	2	-	-	-
Whey	6	-	-	-
Lysine	0.38	0.40	0.26	0.20
Methionine	0.14	0.07	-	-
Threonine	0.12	-	-	-
Calcium carbonate	0.78	0.90	0.75	0.78
Calcium hydrophosphate	0.83	0.58	1.00	0.67
Choline chloride	0.10	0.10	0.10	0.10
Zinc oxide	0.3	-	-	-
Salt	0.30	0.30	0.30	0.30
Premix *	0.35	0.35	0.35	0.35
Total	100	100	100	100
Nutritional compositions, %				
Digestible energy, MJ/kg	3.47	3.34	3.34	3.34
Crude protein	19.54	17.87	16.29	15.11
Ca	0.81	0.67	0.60	0.52
Total P	0.62	0.53	0.52	0.45
Available phosphorus	0.45	0.35	0.33	0.27
Lysine	1.34	1.21	0.97	0.85
sulfur-containing amino acid	0.76	0.65	0.53	0.50
Threonine	0.89	0.73	0.67	0.56

* Supplied the following per kilogram of complete diet: 100 mg of Fe; 15 mg of Cu; 120 mg of Zn; 40 mg of Mn; 0.3 mg of Se; 0.25 mg of I; 12,000 IU of vitamin A; 2250 IU of D3; 24 IU of vitamin E; 6.2 mg of riboflavin; 25 mg of nicotinic acid; 15 mg of pantothenic acid; 1.2 mg of vitamin B_12_; 0.15 mg of biotin.

**Table 3:** Primer sequences of the target and reference genes.

Gene	Primers	Sequence	Accession Number	Product Size (bp)
*gp_X_*	Forward	5′-GCTCGGTGTATGCCTTCTCT-3′	NM_214201.1	103
	Reverse	5′-AGCGACGCTACGTTCTCAAT-3′		
*Gr*	Forward	5′-CTACGTGAGCCGACTGAACA-3′	AY368271.1	146
	Reverse	5′-TCAGGATGTGAGGAGCTGTG-3′		
*CuZn-sod*	Forward	5′-GAGACCTGGGCAATGTGACT-3′	GU944822.1	139
	Reverse	5′-CTGCCCAAGTCATCTGGTTT-3′		
*Mn-sod*	Forward	5′-TGGAGGCCACATCAATCATA-3′	NM_214127.2	136
	Reverse	5′-AGCGGTCAACTTCTCCTTGA-3′		
*e-sod*	Forward	5′-ACGCTGCTCTGTGCTTACCT-3′	NM_001078688.1	142
	Reverse	5′-TCAACTCCTGCCAGATCTCC-3′		
*Aat*	Forward	5’-CTCGGGTGTCACAGAGGAAC-3’	X88780.1	132
	Reverse	5’-GTGGCCTCTGTCCCTTTCTC-3’		
*Gen*	Forward	5’-CACTACTGGCTGGGCAATGA-3’	X13871.1	87
	Reverse	5’-ATCCTGATGCCACTCCTCCT-3’		
*Afp*	Forward	5’-GCACGAAGAGATGCCCATAAAC-3’	NM214317.1	116
	Reverse	5’-ATGTCTCATCCAACACCAAGCT-3’		
*grp78*	Forward	5’-AACCAAGGACGCTGGAACTATT-3’	X92446.1	181
	Reverse	5’-AACACCAGGATGTTCTTCTCCC-3’		
*lhx8*	Forward	5’-GCTGTCTCCACCCATGTTAGAA-3’	FJ587986.1	92
	Reverse	5’-CATCCATGTAACTATGCAGCGC-3’		
*Nobox*	Forward	5’-CCAAGAAGACCACTATCCGGAC-3’	FJ587509.1	150
	Reverse	5’-GTGTCCTTGTTCTCCTTCCCAT-3’		
*sohlh2*	Forward	5’-TTGAACGTGTACTCTGTCCCTG-3’	XM_013980443.1	147
	Reverse	5’-AGCATGGAAGGAATTGAGAGCA-3’		
*bcl2*	Forward	5’-CTTGGAGGGGACACTCTTCTTC-3’	XM_003121700	140
	Reverse	5’-TTTCTCATCACTGTCCTTCGGG-3’		
*Bax*	Forward	5’-TTCAGGGTTTCATCCAGGATCG-3’	XM_003127290	157
	Reverse	5’-ATCCTCTGCAGCTCCATGTTAC-3’		
*cas3*	Forward	5’-TGGCGTGTCAGAAAATACCAGT-3’	NM_214131.1	94
	Reverse	5’-GATCCGTCCTTTGAATTTCGCC-3’		
*β-actin*	Forward	5’-GGCCGCACCACTGGCATTGTCAT-3’	DQ845171.1	104
	Reverse	5’-AGGTCCAGACGCAGGATGGCG-3’		

*gpx*, glutathione peroxidase; *gr*, glutathione reductase; *CuZn-sod*, cytoplasmic copper/zinc superoxide dismutase; *Mn-sod*, manganese superoxide dismutase; *g-sod*, extracellular superoxide dismutase; *aat*, alpha-1-antitrypsin; *gen*, gelsolin; *afp*, alpha-fetoprotein; *grp78*, 78-kDa glucose-regulated protein; *lhx8*, LIM homeobox protein 8; *nobox*, newborn ovary homeobox gene; *sohlh2*, spermatogenesis and oogenesis-specific basic helix-loop-helix 2; *bcl2*, B-cell lymphoma-2; *bax*, Bcl-2 assaciated X protein; *cas3*, caspase-3.

**Table 4:** Effects of maternal high fat nutrition treatments on the weight performance of offspring female gilts at various ages.

Item	CON	HFD	*p*-Value
Body weight at Day 55 of gestation (g)	92.59 ± 2.21	86.07 ± 3.56	0.195
Body weight at Day 90 of gestation (g)	673.34 ± 57.88	632.32 ± 102.17	0.739
Body weight at birth (kg)	1.17 ± 0.69	1.20 ± 0.15	0.859
Body weight at Day 160 of offspring (kg)	75.75 ± 0.85	84.13 ± 3.73	0.071
Body weight upon puberty of offspring (kg)	110.8 ± 2.41	110.8 ± 2.14	1

CON, normal dietary group and gilts were fed diets containing 7.27% crude fat during gestation; HFD, high fat dietary group and gilts were fed diets containing 11.78% crude fat during gestation (*n* = 4 for each group).

**Table 5:** Effect of antenatal high fat nutrition on ovarian and growth performance at Day 160 and upon puberty of offspring gilts.

Item	d160		*p*-Value	Puberty		*p*-Value
	CON	HFD		CON	HFD	
Small follicles numbers (1–3 mm)	13.33 ± 1.20	14.00 ± 3.05	0.849	29.33 ± 1.76	16.33 ± 4.10	<0.05
Large follicles numbers (≥4 mm)	16.67 ± 1.76	9.33 ± 0.67	<0.05	16.67 ± 0.67	14.00 ± 1.00	0.091
Ovary weight (g)	10.18 ± 0.70	9.70 ± 0.05	0.530	16.96 ± 1.07	11.25 ± 2.17	0.078
Liver weight (g)	1403.4 ± 37.00	1241.75 ± 52.78	<0.05	1768.5 ± 43.12	1667.33 ± 84.78	0.299
Spleen weight (g)	158.53 ± 8.80	158.33 ± 17.18	0.186	275 ± 33.29	201.5 ± 9.95	0.059
Back fat thickness (mm)	14.6 ± 0.05	18.2 ± 0.06	<0.05	19.6 ± 0.03	23.9 ± 0.11	<0.05
Age at puberty (day)	-	-	-	185.00 ± 2.04	189.50 ± 3.86	0.370

d160, at Day 160 of the offspring; puberty, upon the offspring reaching puberty; CON, normal dietary group and gilts were fed diets containing 7.27% crude fat during gestation; HFD, high fat dietary group and gilts were fed diets containing 11.78% crude fat during gestation (*n* = 4 for each group).

**Table 6:** Effect of antenatal high fat nutrition on ovarian oxidative status at various offspring ages.

Item	g55		*p*-Value	g90		*p*-Value	d160		*p*-Value	Puberty		*p*-Value
	CON	HFD		CON	HFD		CON	HFD		CON	HFD	
MDA, nmol/mg	6.98 ± 0.14	7.55 ± 0.47	0.315	7.47 ± 0.57	9.57 ± 0.48	<0.05	6.98 ± 0.22	6.42 ± 0.07	0.078	6.57 ± 0.85	4.95 ± 0.25	0.143
T-SOD, U/mg	55.50 ± 0.69	62.42 ± 4.11	0.173	67.72 ± 2.77	57.55 ± 1.08	<0.01	33.06 ± 1.87	25.51 ± 1.34	<0.05	30.58 ± 0.47	33.07 ± 1.46	0.18
CuZn-SOD, U/mg	47.17 ± 3.41	58.16 ± 4.40	0.119	65.52 ± 3.95	53.91 ± 1.67	<0.05	23.61 ± 4.60	27.61 ± 4.39	0.563	25.04 ± 0.59	15.22 ± 1.84	0.143
GR, U/g	22.18 ± 1.70	20.29 ± 0.97	0.389	21.00 ± 0.45	19.27 ± 0.64	0.05	11.39 ± 1.15	13.84 ± 1.47	0.258	14.70 ± 0.72	15.85 ± 0.52	0.265
GPx, U	50.45 ± 4.50	55.00 ± 1.73	0.400	115 ± 3.19	95 ± 2.32	<0.01	127.91 ± 9.51	90.49 ± 2.03	<0.05	100 ± 9.97	146 ± 15.27	0.066
T-AOC, U/mg	11.72 ± 0.52	8.69 ± 0.76	<0.05	9.44 ± 0.59	6.86 ± 0.20	<0.05	5.29 ± 0.22	3.28 ± 0.29	<0.05	6.89 ± 0.22	5.81 ± 0.20	<0.05

g55, at Day 55 of gestation of the fetus; g90, at Day 90 of gestation of the fetus; d160, at Day 160 of offspring; puberty, upon the offspring reaching puberty. CON, normal dietary group and gilts were fed diets containing 7.27% crude fat during of gestation; HFD, high fat dietary group and gilts were fed diets containing 11.78% crude fat during of gestation. MDA, determine malondialdehyde; T-SOD, total superoxide dismutase; CuZn-SOD, cytoplasmic copper/zinc superoxide dismutase; GR, glutathione reductase; GPx, glutathione peroxidase; T-AOC, total antioxidative capability (*n* = 4 for each group, the fetus samples were from 4 gilts, and each gilt provided 2 fetuses; the calculated average values of the two fetal ovaries for each gilt were used in the statistical analysis).
